# Suprachiasmatic to paraventricular nuclei interaction generates normal food searching rhythms in mice

**DOI:** 10.3389/fphys.2022.909795

**Published:** 2022-10-06

**Authors:** Iwona Olejniczak, Benjamin Campbell, Yuan-Chen Tsai, Shiva K. Tyagarajan, Urs Albrecht, Jürgen A. Ripperger

**Affiliations:** ^1^ Department of Biology, Faculty of Science and Medicine, University of Fribourg, Fribourg, Switzerland; ^2^ Institute of Pharmacology and Toxicology, University of Zurich, Zurich, Switzerland

**Keywords:** circadian, homeostat, hedonistic, suprachiasmatic nuclei, GABA

## Abstract

Searching for food follows a well-organized decision process in mammals to take up food only if necessary. Moreover, scavenging is preferred during their activity phase. Various time-dependent regulatory processes have been identified originating from the suprachiasmatic nuclei (SCN), which convert external light information into synchronizing output signals. However, a direct impact of the SCN on the timing of normal food searching has not yet been found. Here, we revisited the function of the SCN to affect when mice look for food. We found that this process was independent of light but modified by the palatability of the food source. Surprisingly, reducing the output from the SCN, in particular from the vasopressin releasing neurons, reduced the amount of scavenging during the early activity phase. The SCN appeared to transmit a signal to the paraventricular nuclei (PVN) *via* GABA receptor A1. Finally, the interaction of SCN and PVN was verified by retrograde transport-mediated complementation. None of the genetic manipulations affected the uptake of more palatable food. The data indicate that the PVN are sufficient to produce blunted food searching rhythms and are responsive to hedonistic feeding. Nevertheless, the search for normal food during the early activity phase is significantly enhanced by the SCN.

## Introduction

The search for food to maintain energy balance is important for the survival of an organism. Because it is such an important process, multiple decision-making mechanisms affect the motivation to scavenge. For example, in mammals a complex system is in place to balance energy intake (Williams et al., 2001; Richard 2007; Konner et al., 2009). Hormonal signals from the periphery such as leptin and insulin indicate a surplus of digested food (Boucsein et al., 2021). The signaling peptides are detected by the proopiomelanocortin (POMC) and neuropeptide Y (NPY) neurons in the arcuate nucleus (Arc) (Luquet et al., 2005), leading to demotivation of food searching behavior (Joly-Amado et al., 2014). The chain of events involves signaling from the Arc to the paraventricular nuclei (PVN) (Atasoy et al., 2012). These nuclei can integrate a multitude of hormonal signals from the Arc and brainstem and represent the main decision center to control the energy balance of an organism (Dos-Santos et al., 2019; Lin and Chen 2019; Herman et al., 2020; Liu et al., 2021). Then the PVN transmit the satiety signal further to activate neurons within the metabolic brainstem to stop the search for food.

On the other hand, the hunger signal ghrelin, which is derived from the empty stomach (Maric et al., 2014), stimulates a different set of sensory cells in the Arc, the agouti-related protein (AgRP) neurons (Sanchez-Lasheras et al., 2010; Joly-Amado et al., 2014). These cells signal to the lateral hypothalamus to repress the neurons within the metabolic brainstem to provoke food-searching behavior. The Arc is located close to a permissive barrier for humoral signals from the periphery, the median eminence (Korf and Moller 2021). The Arc is also in contact with the third ventricle, which is filled with cerebral spinal fluid (CSF) reflecting the nutrition state of the brain (Bolborea et al., 2020). This location could favor the Arc over other brain regions to start the decision-making process maintaining energy homeostasis.

However, the homeostatic component is by far not the only decision process involved. More palatable food triggers the mesolimbic reward system to release dopamine (Zhou and Palmiter 1995; Baik 2021). This strong pleasure signal can overcome the block of food searching imposed by the brainstem. Furthermore, under normal conditions organisms restrict the search for food to their activity phase, when their food is more easily accessible or the risk is reduced of becoming themselves pray (Panda 2016; Challet 2019). The partition in activity and rest phases allows an organism to synchronize its physiology to the external light/dark cycle, which is measured in mammals by a specific center close to the optical chiasm, the suprachiasmatic nuclei (SCN) (Albrecht 2012). The SCN translate specific times of a day into neuronal and humoral output signals. In this context, two subregions of the SCN are worth mentioning. The vasoactive intestinal peptide (Vip) subregion directly receives the light input, while the vasopressin (Avp) subregion governs many of the output functions (Abrahamson and Moore 2001; Vosko et al., 2015; Mieda 2019; Ono et al., 2021b).

Even in the absence of external light information, the SCN maintain an activity rhythm with a period of about a day, which is defined as circadian rhythm. Lesion studies to unravel the functions of the SCN demonstrated an impact on rhythmic food intake (Ishikawa and Shimazu 1980; Stoynev et al., 1982; Janssen et al., 1994; Coomans et al., 2013). However, the superiority of the SCN was challenged by the observation that many peripheral oscillators, while under normal conditions being responsive to SCN signaling, could be uncoupled from this signaling by inverted feeding (Damiola et al., 2000; Stokkan et al., 2001). Consequently, it was supposed that the SCN maintain a food searching rhythm which would then synchronize the peripheral oscillators by the time of food uptake (Stephan 2002; Schibler et al., 2003).

Meanwhile, more direct functions of the SCN on food uptake have been identified. Upon shining light on rats at the beginning of their activity phase in the dark, the animals stopped eating and moving (Santoso et al., 2018). This light pulse caused the release of vasopressin from the Avp region of the SCN. The target appeared to be oxytocin-releasing neurons in the PVN. The inhibition of these particular neurons in the PVN may affect POMC neurons in the nucleus tractus solitarius of the brainstem (Maejima et al., 2009). The activation of these neurons could then stop the food uptake and movement of the rats. A completely different mechanism was recently described for the interaction of the SCN with the Arc region (Rodriguez-Cortes et al., 2022). In this scenario, at the time of transition to the rest phase, vasopressin released from the SCN increased the transport of glucose into the CSF of the third ventricle. This increase of glucose was then sensed by the Arc–probably *via* a subset of POMC neurons–to lower the blood glucose concentration in the periphery. At the transition to the activity phase, the process was reversed. Overall, the glucose concentration in the blood is rhythmic with slight elevation during the activity phase, and slight reduction during the rest phase to balance the required needs. Taken together, the SCN may affect more than just setting the time of food uptake.

The neuronal activity of the SCN of mice prevails during the rest phase (Belle et al., 2009; Atkinson et al., 2011; Belle and Diekman 2018). However, the different subregions of the SCN allow for the regulation of output during the entire circadian cycle. For example, Vip neurons regulate the time of the siesta, a short sleeping period amidst the end of the activity phase of a mouse (Collins et al., 2020), or impact the activity of the PVN to regulate corticosterone secretion (Jones et al., 2021). On the other hand, Avp neurons in the SCN promote water uptake at the transition to the rest phase to prevent dehydration (Gizowski et al., 2016), while a couple of hours earlier the same neurons prevent dehydration by responding to an increase of the sodium ion concentration in the CSF (Gizowski and Bourque 2020). Hence, the SCN are predestined to regulate time-of-day-dependent processes.

Due to the manifold functions of the SCN, we wanted to revisit its impact on the normal food uptake of mice. To get new insights, we developed a food searching assay, and used genetic tools to block connections between neighboring brain regions. In particular, we focused on the potential interaction between the SCN and the PVN, which would combine the circadian with the energy balancing brain centers to optimize the time of food uptake.

## Materials and methods

### Animals

**FIGURE 1:**
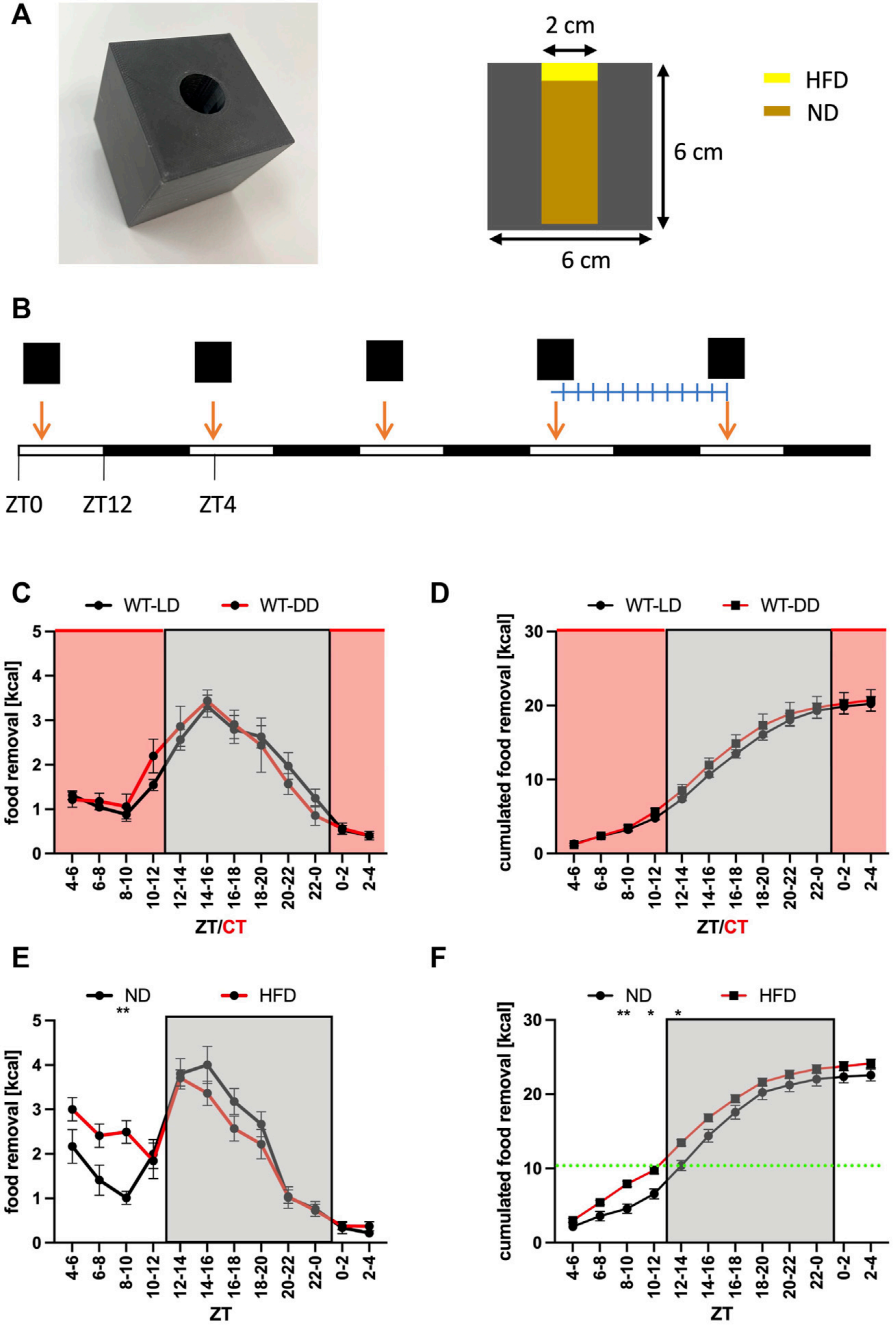
Normal food removal is not affected by the presence of light. **(A)** Picture and dimensions of the feeding cube. The animals have access to food *ad libitum*. Note that in some experiments high-fat diet was placed on top of the hole. **(B)** Outline of the experiment. The cube is replenished every day at ZT4. After an entrainment period, the weight of the box is determined every 2 h. **(C)** Food removal in each 2 h bin starting from ZT4-6 for WT in 12 h light/12 h dark (LD, black) or 12 h dark/12 h dark (DD, red) conditions (2-way repeated measure ANOVA, N = 12, *p* = 0.8, F = 0.06, DFn = 1, DFd = 22). **(D)** Accumulation of food removal over the course of the experiment 1C (2-way repeated measure ANOVA, N = 12, *p* = 0.51, F = 0.44, DFn = 1, DFd = 22). **(E)** Food removal in each 2 h bin starting from ZT4-6 for WT with normal diet (black) or high-fat diet (red) (2-way repeated measure ANOVA, N = 12, *p* = 0.85, F = 0.03, DFn = 1, DFd = 22). **(F)** Accumulation of food removal over the course of the experiment 1E (2-way repeated measure ANOVA, N = 12, *p* = 0.82, F = 0.05, DFn = 1, DFd = 22). A green line indicates the transition from HFD to ND. To correct for multiple testing, a Šidák’s post hoc test was employed; *: *p* < 0.05; **: *p* < 0.01. Shown is the mean ± SEM.

Prior to the experiments the animals were housed in groups of up to 4 in Techniplast Makrolon type 2 cages with stainless-steel wire lids (No. 1264C001 and 1264C116; Techniplast, Hohenpreissenberg, Germany) with food and water *ad libitum*. During the experiments, the animals were single-housed in a cage equipped with a running wheel (Jud et al., 2005) with the content of the feeding cube as an *ad libitum* food source (Figures 1A,B). We used male C57BL/6JRj wild-type animals (Janvier Labs, Le Genest-Saint-Isle, France) aged 2–3 months. The Vip-Cre mice (B6J.Cg-*Vip* ^*tm1(cre)Zjh*^/AreckJ; strain #:031628; The Jackson Laboratory, Bar Harbor, ME, United States) (NIH Neuroscience Blueprint Cre Driver Network 2009), and the Avp-Cre mice (B6J.Cg-*Avp* ^*tm1.1(cre)Hze*^/J; strain #:023,530; The Jackson Laboratory, Bar Harbor, ME United States) (Daigle et al., 2018) were maintained and used as heterozygous alleles. Furthermore, we used *Gabra1 floxed/floxed* mice (B6.129(FVB)-*Gabra1* ^*tm1Geh*^/J; strain #:004,318; The Jackson Laboratory, Bar Harbor, ME, United States) (Kralic et al., 2006) and *Gabra2 floxed/floxed* mice (Gabra2^tm2.1Uru^; MGI # 3614426) (Panzanelli et al., 2011). Housing as well as experimental procedures were performed in accordance with the guidelines of the Schweizer Tierschutzgesetz and the Declaration of Helsinki. The Animal experimentation commission of the Canton of Fribourg approved the protocols (2019-35-FR, national number 31873). The study was carried out in compliance with the ARRIVE guidelines.

### Adeno-associated viral vectors

All adeno-associated viral vectors (AAV) were purchased from the Viral Vector Facility of the University of Zurich and ETH (https://www.vvf.uzh.ch). We used the following vectors: v150 (ssAAV-capsid 9/2-hSyn1-chI-mCherry_ 2A_FLPo-WPRE-SV40pA) to express FLP recombinase and Cherry in neurons, v150 (ssAAV-capsid R/2-hSyn1-chI-mCherry_2A_FLPo-WPRE-SV40pA) with the ability of retrograde transport and expression of FLP recombinase and Cherry in upstream neurons, v229 (ssAAV-capsid 9/2-hSyn1-EGFP_iCre-WPRE-hGHpA) to express Cre recombinase and eGFP in neurons, v450 (ssAAV-capsid 9/2-hSyn1-chI-dFRT-EGFP_2A_FLAG_TeTxLC (rev)-dFRT-WPRE-hGHpA to express eGFP and tetanus toxin light chain upon reversion by FLP recombinase, and v322 (ssAAV-capsid 9/2-hSyn1-chI-dlox-EGFP_2A_FLAG_TeTxLC (rev)-dlox-WPRE-SV40pA) to express eGFP and tetanus toxin light chain upon reversion by Cre recombinase.

### Preparation of the feeding cubes and food removal assay

The feeding cubes were 3D-printed using a Zortrax M200 Plus printer and Z-ESD plastic material (Zortrax, Olsztyn, Poland). The dimensions are indicated in (Figure 1A). Unfortunately, this cube could not be used for the work with female mice, which were able to remove the entire food in 1 day (10–11 g corresponding to half of their body weight). We suspect that the opening of the cube (2 cm in diameter) was too large for the female mice and it was too easy for them to remove the entire content. Hence, we were forced to restrict our analysis to male mice. Normal diet (1 g = 3.15 kcal; 3432, Kliba Nafag, Kaiseraugst, Switzerland) was ground with a kitchen mixer to a fine powder and stuffed into the cavity of the cube. The food was compressed to make it not too easy to remove it from the cube. High-fat diet with 60% of the energy coming from fat (1 g = 4.8 kcal; 2127, Kliba Nafag, Kaiseraugst, Switzerland) was put like a chewing gum to seal the top of the opening. To prevent problems with overeating due to the addictive-like character of this kind of diet, we restricted the amount of high-fat diet to about 50% of the amount removed normally by mice (defined as kcal content of standard chow removed from the feeding cube at baseline). Animals were entrained to the feeding protocol for 3–5 days (Figure 1B). The day before the experiment, the cages were cleaned and supplemented with new litter and nesting material. The day of the experiment, the feeding cubes were weighed to 0.01-gram precision. Each cube was given at ZT4 and then every 2 h the weight of the cube measured to determine the removal of food in 2 h bins. Data were filled into a preformatted EXCEL sheet (Microsoft, Redmond, MA, United States) and analyzed with Prism9 (GraphPad software, La Jolla, CA, United States).

### Stereotactic brain injections

2-month-old mice were selected for the stereotaxic injections. The animals received a subcutaneous injection of buprenorphine (Temgesic^®^, Indivior, Switzerland; 0.1 mg/kg body weight) and carprofen (Rimadyl^®^, Zoetis, Switzerland; 5 mg/kg body weight) half an hour before the procedure. Then, the animals were anesthetized with isoflurane (induction with 5.0% v/v, 1 l O_2_/min, and maintained at 2.5% isoflurane v/v, 1 l O_2_/min) and placed in a stereotaxic frame (Stoelting Co., Wood Dale, IL, United States). A small area on top of the head was shaved and sterilized with betadine solution (Mundipharma, Switzerland). To prevent dehydration, a drop of Lacryvisc (Alcon, Switzerland) was applied to each eye for multiple times during the operation. An 0.5 cm incision into the skin was made on the top of the skull and the incision treated with 2% Lidocain/0.9% NaCl (Streuli, Switzerland) as additional local analgesic. Then, the region was sterilized with 3% hydrogen peroxide, and 2 holes were drilled into the skull to allow the descent of a needle. The bregma was used as reference point for the coordinates (set to position 0, 0, 0). The injection was performed with an Ultra precise digital dual injection device (Stoelting Co., Wood Dale, IL, United States) using a pulled glass pipette (10µl glass micropipette, Cat number: 5-000-1001-X10, Thermo Fisher Scientific Inc., Waltham, MA, United States) attached to a hydraulic manipulator (MO-10 one-axis oil hydraulic micromanipulator, Narishige, Tokyo, Japan). For the SCN, two injections of 100 nl each occurred at 0.35 mm posterior to the bregma, 1.83 mm lateral to the midline, and 5.65 mm ventral to the skull surface with an angle of 15°. For the PVN, two injections of 200 nl each occurred at 1.00 mm posterior to the bregma, 0.40 mm lateral to the midline, and 4.90 mm ventral to the skull surface. The injection speed was 40 nl/min. After injection, the glass pipette was risen by 0.1 mm and kept in place for 5 min to prevent upward spread of the virus. After the surgery, the mice received an intraperitoneal injection of 0.4 ml 0.9% NaCl to compensate for dehydration, and the mice were left to awake. The mice were allowed to recover for 2 weeks before the next step of the experiment and carprofen (Rimadyl^®^, Zoetis, Switzerland; 5 mg/kg body weight) was given once a day subcutaneously as analgesia after surgery for 2 more days.

### Animal numbers and surgery exclusion criteria

Based on pilot experiments, we estimated to observe a big effect (effect strength about 0.8) between control and experimental mice on food searching. Considering an alpha error of 0.05, a correlation among repeated measures of 0.4, 2 groups and 12 measurements per animal, we calculated a sample size of 12 animals yielding an actual power of 0.96. Hence, we used 12 control animals and 12 experimental animals for each experiment. Hypothetical exclusion criteria for the use of animals after surgery were based on a score sheet monitoring health parameters of the animal. No animal had to be excluded based on these criteria. Because individual food searching patterns of mice are quite variable even within a given group, exclusion criteria for the actual experiment were not based on the measured food searching pattern. Hence, the food searching patterns of all 12 animals were combined for the analysis albeit of the severity of the phenotype.

### Immunofluorescence

The animals were deeply anaesthetized by intraperitoneal injection of ketamine (Narketan^®^, Vetoquinol AG, Switzerland; 133 mg/kg) and medetomidine (Domitor^®^, Provet AG, Switzerland; 0.3 mg/kg), and perfused intracardially with 10 ml of 0.9% NaCl and 25 ml of 4% paraformaldehyde in 1 x phosphate-buffered saline (PBS). The brain was transferred to a glass vial and post-fixed 16 h (only 2 h for the detection of Gabra1 and Gabra2) at 4°C in 4% paraformaldehyde in 150 mM phosphate-buffered saline (PBS). Then the brain was incubated in 30% sucrose/1 x PBS at 4°C until it sank down to the bottom of the glass vial (16 h +). The brain was cut to 30 μm slices using a Microm HM 550 cryostat (Histocom AG, Zug, Switzerland), or, for the detection of Gabra1 and Gabra2, to 40 μm slices using a Microm HM 400 sliding microtome (Histocom AG, Zug, Switzerland). The immunofluorescence experiments were performed as described (Notter et al., 2014; Olejniczak et al., 2021). We used anti-Neurophysin 2/NP-Avp (clone PS45, MABN856, Merck, Darmstadt, Germany) to detect Avp-positive tissue, and anti-Vip (Abcam30680, Abcam, Cambridge, United Kingdom) to detect Vip-positive tissue. The antibodies against Gabra1 and Gabra2 have been described (Fritschy et al., 2006; Panzanelli et al., 2011).

### Statistical analysis

For statistical analysis of our data, we used Prism9 (GraphPad software, La Jolla, CA, United States). We used 2-way repeated measures ANOVA both for the analysis of food removal bins between control and experiment, and the corresponding accumulation of food removal over time. For both types of analyses, we used Šidák’s post hoc test to correct for the multiple testing of the means of the two bins for each time point. For all experiments we used 12 animals for the experiment and 12 animals for the control, and calculated the mean and the standard error of the mean. *: *p* < 0.05; **: *p* < 0.01; ***: *p* < 0.001.

## Results

### The search for food in mice depends on the food source

To get insights into the function of the SCN for food searching in mice, we performed a food removal assay using a feeding cube (Figure 1A). Mice were given the cube at ZT4 (Zeitgeber time, when ZT0 = light on, and ZT12 = light off) and the removal of food was measured after 3 days in 2-h bins (Figure 1B). A plot of the data over the day indicated that most of the food was removed during the activity phase with a rapid onset shortly before the lights were turned off (Figure 1C). Performing the experiment under constant darkness did not affect the time of food removal nor the amount removed indicating that light did not have a suppressing effect on food removal during the rest phase (Figures 1C,D). Consequently, the food searching pattern was not shaped by external light/dark cues. After the establishment of this baseline feeding curve, we gave the mice access to more palatable high fat diet (HFD) (Figures 1E,F). We chose to provide the mice with about one-half of the amount of daily caloric intake worth of HFD to avoid over-eating of the mice with harmful metabolic consequences (Coccurello and Maccarrone 2018; Baik 2021). Due to the construction of the feeding cubes (Figure 1A), the mice had to eat first the HFD before having access to normal diet (ND) *ad libitum*. The mice with access to HFD removed about 30% more calories during the rest phase but the mice on ND were catching up during the activity phase (Figure 1E). Hence, there was no significant increase of total calories removed from the feeding cube over the entire day when consuming HFD (ZT2-4 bin, Figure 1F), indicating that the homeostatic component of food uptake was compensating for the increased caloric uptake during the rest phase. The experiment showed that the food searching rhythm followed a circadian pattern and could be altered by changing the attractiveness of the food source.

### Reducing the neuronal output from the SCN reduces the search for food

**FIGURE 2:**
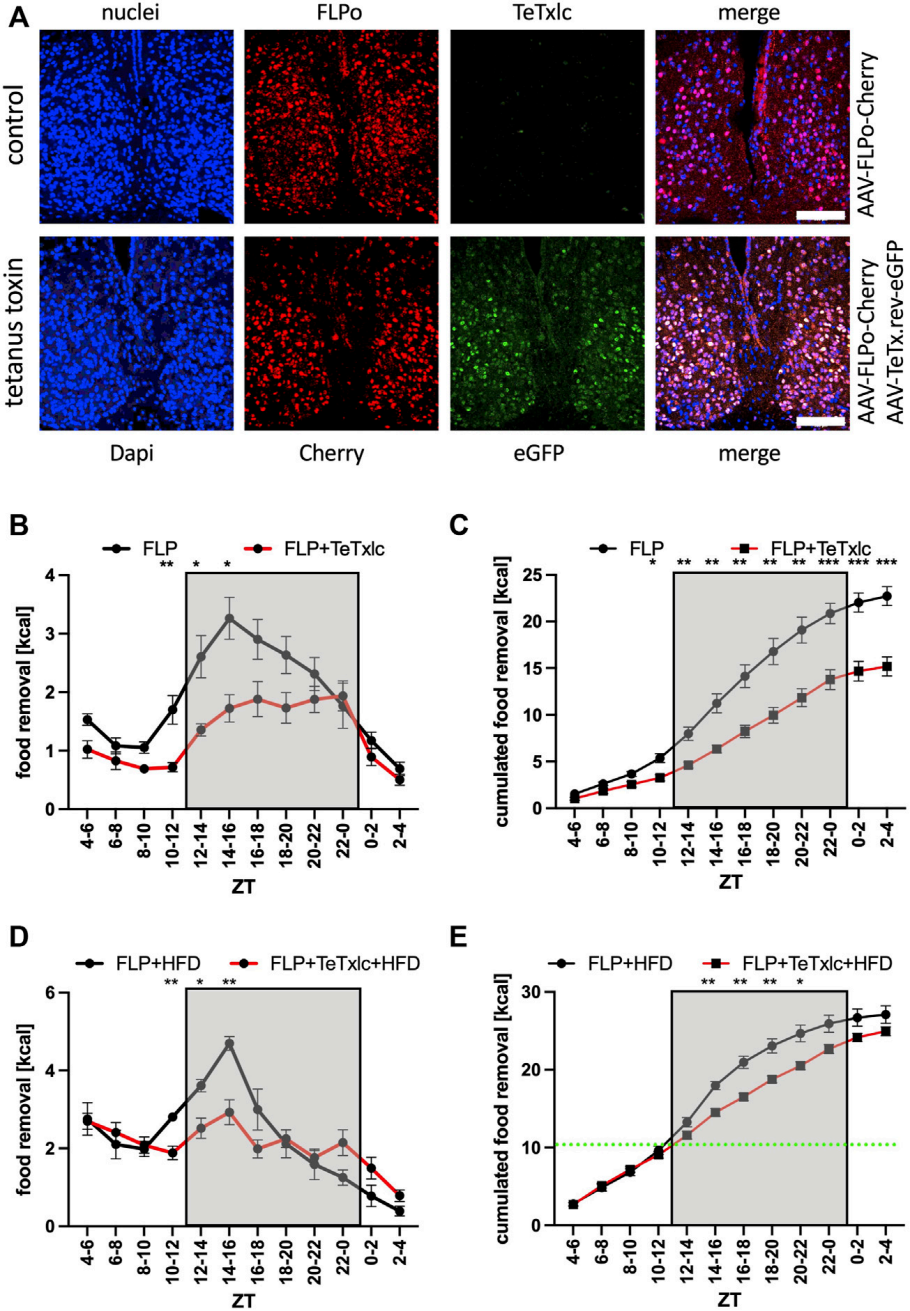
Food removal during the early activity phase is reduced by blocking vesicle release from SCN neurons expressing the tetanus toxin light chain. **(A)** Localization of Cherry (red) and eGFP (green) after injection into the SCN. ×40 objective (numerical aperture = 1.3, oil), a white bar measures 100 μm. **(B)** Food removal in each 2 h bin starting from ZT4-6 for control mice (black), or mice injected with FLP and reversed TeTxlc (red) into both SCN regions (2-way repeated measure ANOVA, N = 12, *p* < 0.001, F = 27.61, DFn = 1, DFd = 22). **(C)** Accumulation of food removal over the course of the experiment 2B (2-way repeated measure ANOVA, N = 12, *p* < 0.001, F = 23.22, DFn = 1, DFd = 22). **(D)** Food removal of control mice (black), or mice injected with FLP and reversed TeTxlc (red) into both SCN regions with high-fat diet (2-way repeated measure ANOVA, N = 12, *p* < 0.01, F = 8.97, DFn = 1, DFd = 22). A green line indicates the transition from high-fat to normal diet. **(E)** Accumulation of food removal over the course of the experiment 2D (2-way repeated measure ANOVA, N = 12, *p* < 0.001, F = 23.22, DFn = 1, DFd = 22). To correct for multiple testing, a Šidák’s post hoc test was employed; *: *p* < 0.05; **: *p* < 0.01; ***: *p* < 0.001. Shown is the mean ± SEM.

Next, we wanted to address the potential function of the SCN on food searching in a more direct fashion. The light chain of tetanus toxin (TeTxlc) can be employed as a tool to reduce synaptic neurotransmitter secretion from neurons. The toxin cleaves the protein VAMP2 located at the outside of synaptic vesicles, which renders the vesicles incapable of Ca^2+^ evoked neurotransmitter release. We injected an adenovirus associated viral (AAV) vector into both sides of the SCN (Figure 2A). The vector expressed FLP recombinase and the fluorescent protein Cherry. To reduce neurotransmitter release, we co-injected an AAV with an inverted version of TeTxlc and eGFP, which needed the help of the FLP recombinase to become active. In the presence of FLP recombinase the toxin and eGFP were activated and we observed a significant reduction of food uptake during the activity phase of the mice (Figures 2B,C). A similar reduction may also occur during the rest phase but the food removal during this time period is quite low, rendering it difficult to observe significant changes. In total, there was an about 25% reduction of calories consumed over the day due to the expression of the toxin (ZT2-4 bin, Figure 2C). Giving the mice access to HFD somewhat neutralized the effect of the block of neurotransmitter release by the expression of tetanus toxin light chain (Figures 2D,E). Both types of mice essentially ate the same amount of HFD in the same time interval (ZT4 to ZT12, green line in Figure 2E), indicating that blocking the SCN did not affect hedonistic feeding. However, the difference in food removal observed at the early activity phase was still observable. Overall, it was a rather strange finding that a functional SCN could promote food removal during the early and middle activity phase, a time during which the neuronal activity of the SCN of mice is reduced (Belle et al., 2009; Atkinson et al., 2011; Belle and Diekman 2018).

### Avp but not Vip neurons mediate the stimulation for food searching

**FIGURE 3:**
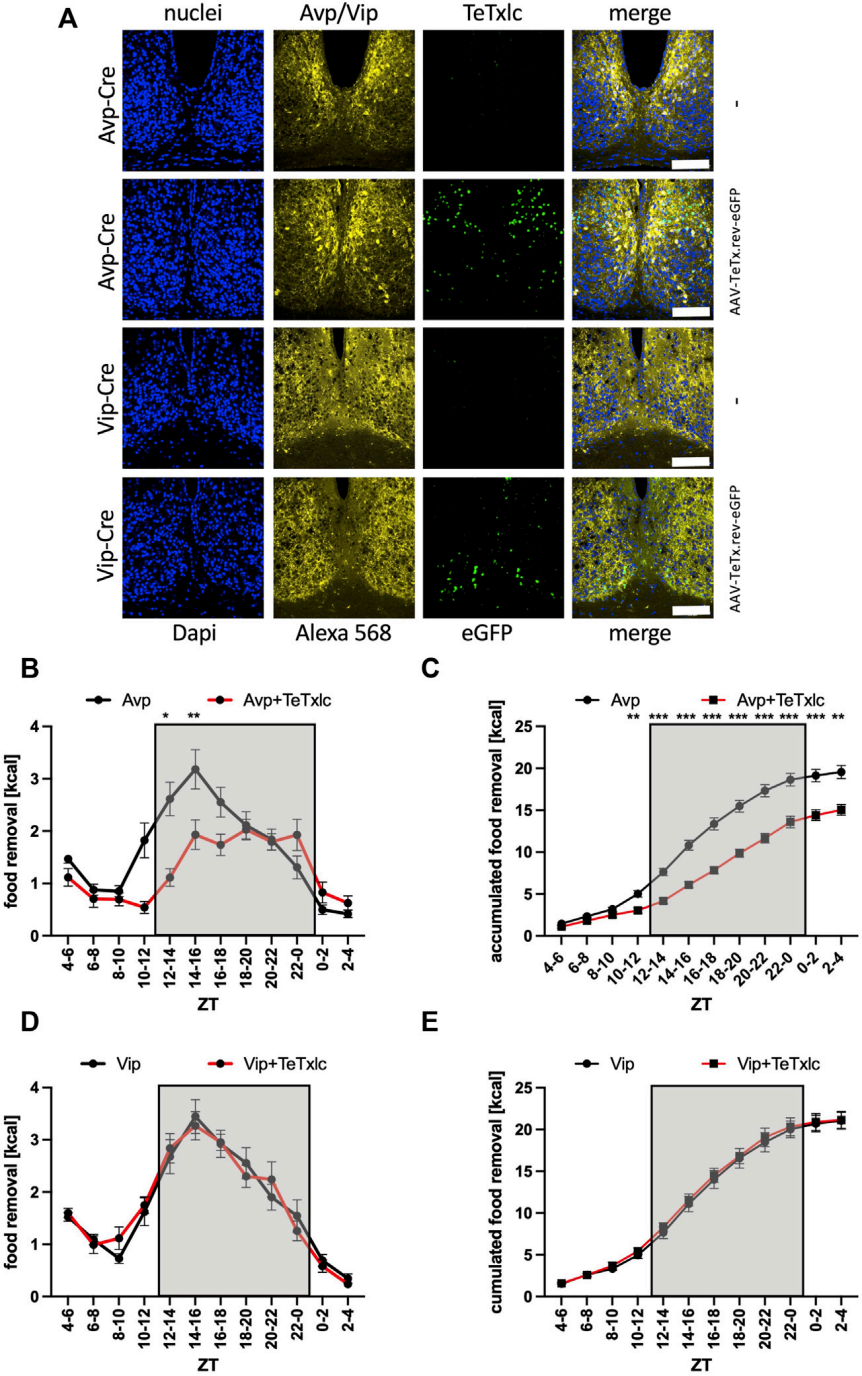
Increase of food removal during the early activity phase is mediated by the Avp subregion. **(A)** Immunofluorescence staining of the localization of Avp or Vip within the SCN (Alexa 568, yellow) and detection of eGFP (green) within the Avp or Vip subregions. ×40 objective (numerical aperture = 1.3, oil), a white bar measures 100 μm. **(B)** Food removal in each 2 h bin starting from ZT4-6 for Avp-Cre mice (black) or Avp-Cre mice injected with reversed TeTxlc (red) into the SCN (2-way repeated measure ANOVA, N = 12, *p* < 0.001, F = 20.42, DFn = 1, DFd = 22). **(C)** Accumulation of food removal over the course of the experiment 3B (2-way repeated measure ANOVA, N = 12, *p* < 0.001, F = 38.85, DFn = 1, DFd = 22). **(D)** Food removal in each 2 h bin starting from ZT4-6 for Vip-Cre mice (black) or Vip-Cre mice injected with reversed TeTxlc (red) into the SCN (2-way repeated measure ANOVA, N = 12, *p* = 0.95, F = 0.00372, DFn = 1, DFd = 22). **(E)** Accumulation of food removal over the course of the experiment 3D (2-way repeated measure ANOVA, N = 12, *p* = 0.73, F = 0.12, DFn = 1, DFd = 22). To correct for multiple testing, the Šidák’s post hoc test was employed; *: *p* < 0.05; **: *p* < 0.01; ***: *p* < 0.001. Shown is the mean ± SEM.

To identify, which subregion of the SCN was responsible for the observed phenotype, we employed mouse strains with expression of Cre recombinase in either Avp or Vip expressing neurons in the SCN to express the tetanus toxin light chain in specific locations (eGFP signal, Figure 3A, Supplementary Figure S1). Without the injection of the toxin-bearing vector, the Avp-Cre mice showed a normal food searching profile (Figures 3B,C). However, the activation of TeTxlc in the Avp subregion of the SCN provoked a significant reduction of food removal, similar to what was observed before for the expression of the toxin in the entire SCN (ZT2-4 bin, Figures 2B,C). By contrast, injecting the same TeTxlc construct into the SCN of mice expressing Cre recombinase in the Vip subregion did not show major differences compared to noninjected Vip-Cre mice (Figures 3D,E). Hence, the motivation to remove food during the activity phase appears to be mediated at least in part by the Avp subregion of the SCN. This scenario somewhat contradicts the action of the same Avp neurons to stop the search for food in response to a light pulse also during the early activity phase (Santoso et al., 2018). However, this effect was dependent on solely the release of vasopressin. We noted that expression of the tetanus toxin light chain did affect neither the expression or accumulation of Avp and Vip (Alexa 568 panels, Figure 3A). Hence, another neurotransmitter secreted from the SCN could impact on the time of food searching.

### Removing Gabra1 but not Gabra2 from the PVN causes a reduction of food searching

**FIGURE 4:**
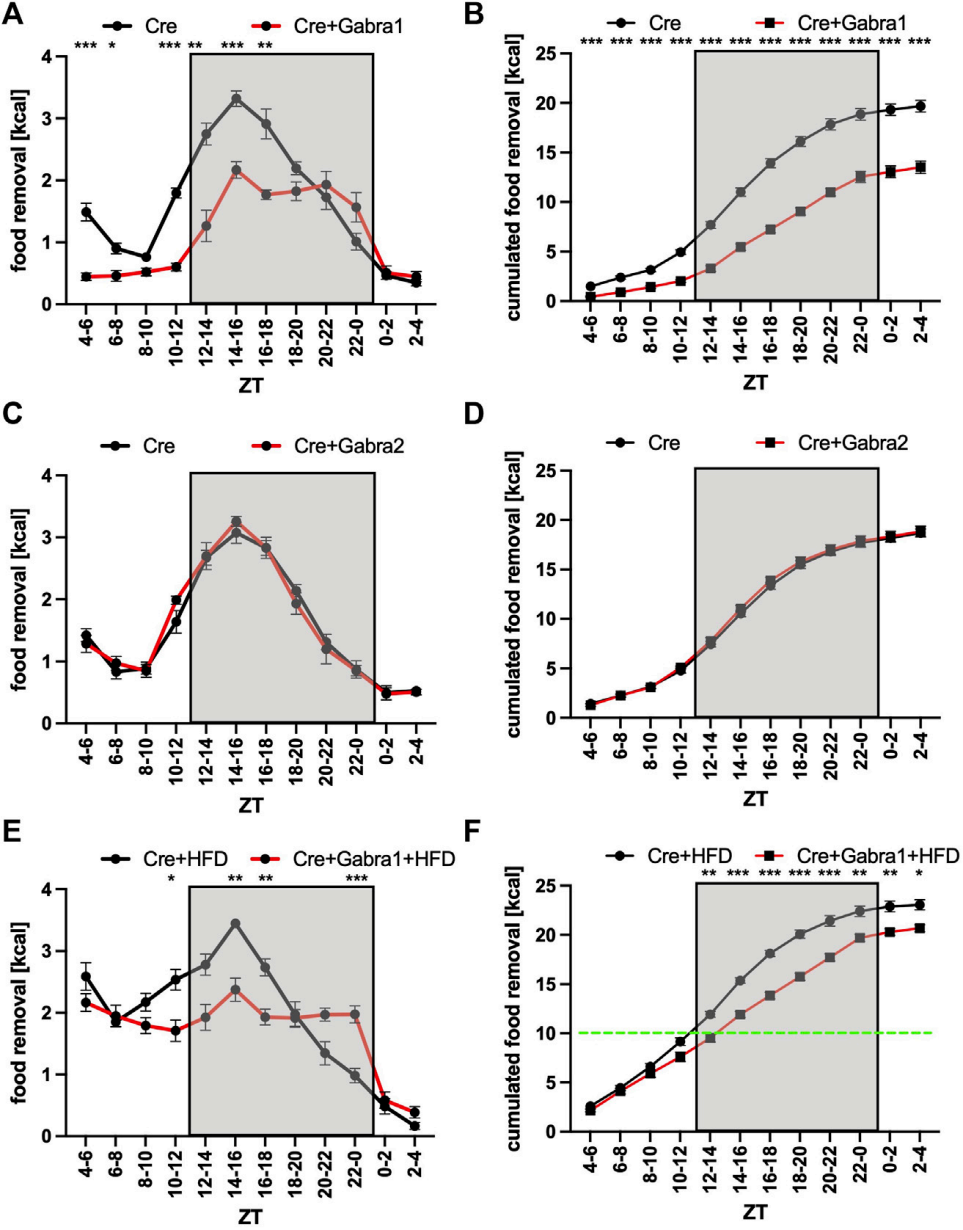
Increase of food removal during the early activity phase relies on Gabra1 in the PVN region. **(A)** Food removal in each 2 h bin starting from ZT4-6 for WT (black) or Gabra1 floxed/floxed (red) mice injected with Cre into both PVN regions (2-way repeated measure ANOVA, N = 12, *p* < 0.001, F = 51.85, DFn = 1, DFd = 22). **(B)** Accumulation of food removal over the course of the experiment 4A (2-way repeated measure ANOVA, N = 12, *p* < 0.001, F = 103.95, DFn = 1, DFd = 22). **(C)** Food removal in each 2 h bin starting from ZT4-6 for WT (black) or Gabra2 floxed/floxed (red) mice injected with Cre into both PVN regions (2-way repeated measure ANOVA, N = 12, *p* = 0.83, F = 0.05, DFn = 1, DFd = 22). **(D)** Accumulation of food removal over the course of the experiment 4C (2-way repeated measure ANOVA, N = 12, *p* = 0.61, F = 0.27, DFn = 1, DFd = 22). **(E)** Same mice as **(A)** but with high-fat diet (2-way repeated measure ANOVA, N = 12, *p* = 0.001, F = 14.23, DFn = 1, DFd = 22). **(F)** Accumulation of food removal over the course of the experiment 4E (2-way repeated measure ANOVA, N = 12, *p* < 0.001, F = 32.50, DFn = 1, DFd = 22). A green line indicates the transition from high-fat to normal diet. To correct for multiple testing, the Šidák’s post hoc test was employed; *: *p* < 0.05; **: *p* < 0.01; ***: *p* < 0.001. Shown is the mean ± SEM.

Except for Avp and Vip, gamma-aminobutyric acid (GABA) is secreted from the SCN (Ono et al., 2021a; Maejima et al., 2021). Hence, we were looking for a way to block a potential GABA signal originating from the SCN on the PVN side, and decided to remove two different kinds of GABA receptors from the PVN. To this intend, we used mouse strains with floxed alleles of either *Gabra1* or *Gabra2* and injected an AAV expressing Cre recombinase into the PVN region (Supplementary Figure S2). Surprisingly, the removal of Gabra1 from the PVN resulted into a nearly identical phenotype as the blocking of neurotransmitter release from the SCN (Figures 4A,B). The food removal from the feeding box was reduced during the activity and rest phase. On the other hand, the same experiment performed with *Gabra2*-floxed mice did not reveal an impact on the time of food removal (Figures 4C,D). Furthermore, to test if the mice can still respond to a more palatable food source, we gave the *Gabra1*-floxed mice access to HFD (Figures 4E,F). As can be seen, HFD could overcome the removal of Gabra1 and the mice removed HFD from the point, when it was given until the beginning of the activity phase (green line, Figure 4F), when all of the HFD was consumed and only normal diet left. Similar to the previous experiments, upon switching to normal diet a significant difference in food removal was observed at the beginning of the activity phase between the mice (Figures 4E,F). This result would indicate that hedonistic food signals are not affected by removing the Gabra1 receptor from the PVN. Taken together, it is possible to block the augmentation of food removal either on the side of the SCN by reducing the release of neurotransmitters using the expression of tetanus toxin light chain, or on the PVN side by removing the GABA receptor A1.

### Direct SCN to PVN interaction is important for food searching

**FIGURE 5:**
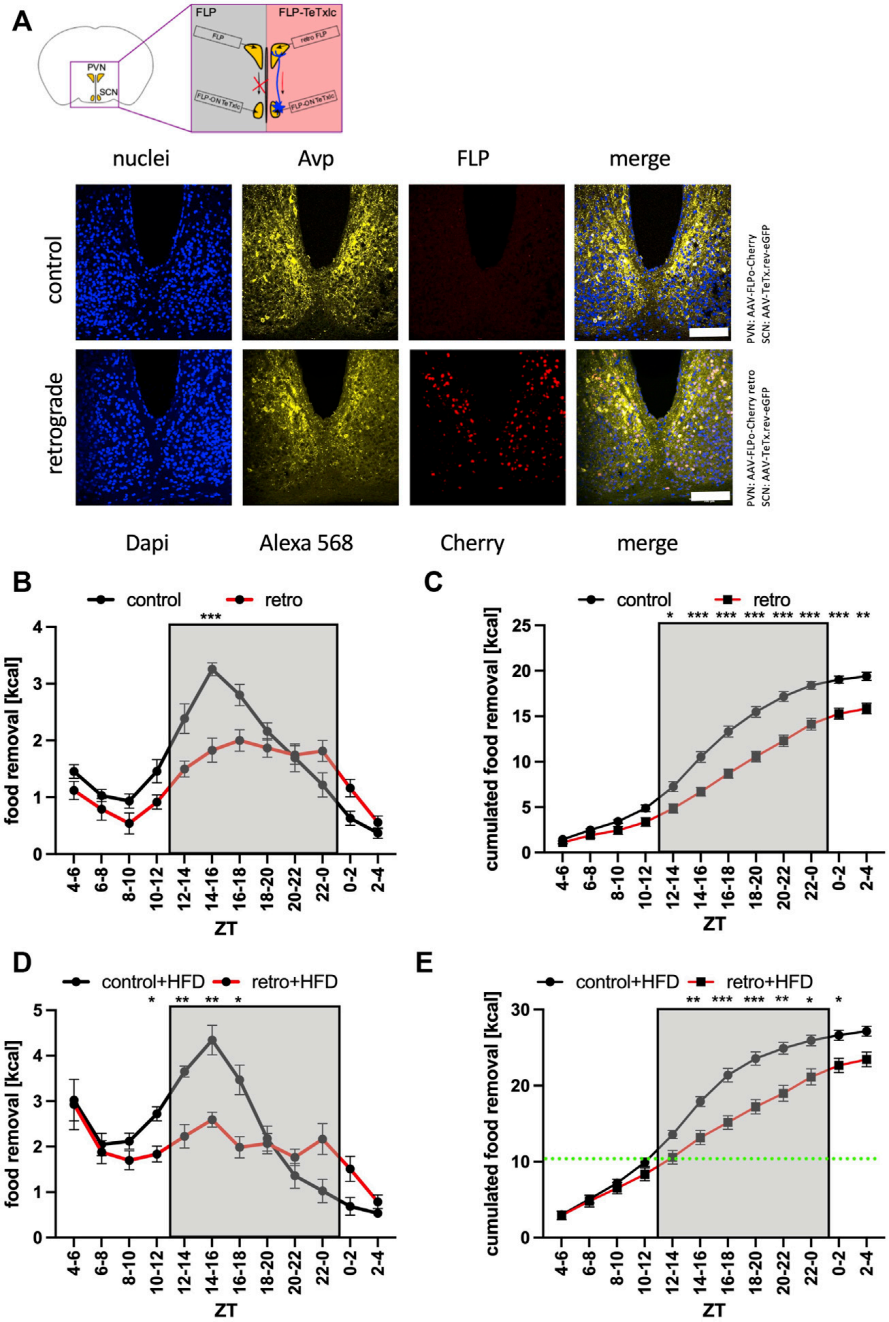
Increase of food removal during the early activity phase relies on the connection between the SCN and PVN regions. **(A)** Outline of the retrograde complementation assay and immunofluorescence staining of Avp (Alexa 568, yellow) and detection of Cherry (red). ×40 objective (numerical aperture = 1.3, oil), a white bar measures 100 μm. **(B)** Food removal in each 2 h bin starting from ZT4-6 for FLP delivered with AAV-9 (control, black) or FLP delivered with AAV-R (retro, red) into both PVN regions, and TeTxlc injected into both SCN regions (2-way repeated measure ANOVA, N = 12, *p* < 0.001, F = 23.48, DFn = 1, DFd = 22). **(C)** Accumulation of food removal over the course of the experiment 5B (2-way repeated measure ANOVA, N = 12, *p* < 0.001, F = 30.06, DFn = 1, DFd = 22). **(D)** Food removal in each 2 h bin starting from ZT4-6 for FLP delivered with AAV-9 (control, black) or FLP delivered with AAV-R (retro, red) into both PVN regions (2-way repeated measure ANOVA, N = 12, *p* < 0.01, F = 8.21, DFn = 1, DFd = 22). **(E)** Accumulation of food removal over the course of the experiment 5D (2-way repeated measure ANOVA, N = 12, *p* = 0.05, F = 4.18, DFn = 1, DFd = 22). To correct for multiple testing, the Šidák’s post hoc test was employed; *: *p* < 0.05; **: *p* < 0.01; ***: *p* < 0.001. Shown is the mean ± SEM.

To gather evidence for a direct interaction of both brain regions, we decided to employ retrograde-mediated complementation as the method of choice. In this approach, the inverted TeTxlc construct and the FLP recombinase were injected into two different brain regions (Figure 5A). Expression of the FLP recombinase occurs either only in the PVN region and is therefore physically separated from the toxin (control), or–using a different capsid protein–the AAV can *via* retrograde transport infect neurons that are upstream of the neurons from the injection site including the SCN (PVN: Supplementary Figure S3, SCN: Figure 5A). Interestingly, retrograde-mediated expression of Cherry and consequently of the FLP recombinase in the SCN was mainly in the Avp region. Testing the control revealed a rather generic food removal pattern similar to untreated mice (Figures 5B,C). Employing the retrograde FLP AAV reduced the amount of food removal similar as expressing the activated toxin in the SCN or Avp subregion (Figures 2B,C, 3B,C). Since the retrograde transport is limited to the next adherent cells upstream of the injection site, this experiment supported the idea of a direct contact between the SCN neurons and their targets in the PVN. As already observed for the previous experiments (Figures 2–4), the access of the mice to HFD could circumvent the block of the SCN neurons in direct contact with the PVN (Figures 5D,E). Thus, the interaction of the SCN with the PVN is important to motivate mice to search for normal but not hedonistic food in the early activity phase.

## Discussion

Animals spend a substantial amount of time searching for food. Consequently, it is not surprising that a manifold of regulatory decisions compound to initiate the food searching process only if necessary. It appears that the most important trigger to search for food initiates from the homeostat system located in the Arc region, which integrates the signals from the periphery (Luquet et al., 2005; Konner et al., 2009; Joly-Amado et al., 2014). The Arc region has been shown to have its own functional circadian system (Cedernaes et al., 2019). It is also indirectly influenced by signals from the SCN, which changed the glucose concentration in the CSF to adapt glucose levels to the daily needs (Rodriguez-Cortes et al., 2022). We found that the homeostatic component of feeding contributed a substantial amount of the signal to search for food, which became apparent if we blocked the SCN to PVN communication (Figures 2–5). Hence, the contribution of the homeostat system may use a different route. It may come from the direct connection of AgRP neurons in the Arc to oxytocin neurons in the PVN (Atasoy et al., 2012). It was previously shown that this connection affected food searching. Another conclusion from our experiments was that light did not drastically affect the homeostat system (Figures 1C,D). This makes sense, because most animals in the field do not live under *ad libitum* conditions and have to take any opportunity to replenish their energy resources independently of the presence or absence of light. In the absence of light, the food search rhythm was slightly advanced indicating free-running conditions governed by the circadian clock (Figures 1C,D). It will be important to investigate this light response in mice with inactivated SCN to PVN connection to determine, whether their food searching pattern was shaped simply by the presence of light during the rest phase, a phenomenon called masking (Albrecht 2012).

Inhibitory signals from the homeostat system can be overcome by dopamine release from the reward system and these may be linked to the circadian clock (Mendoza 2019a). Another conclusion from our experiments was that the hedonistic system did not act *via* the same SCN to PVN connection as described in this paper (Figures 2–5). More palatable food somehow circumvented the block of the SCN or the GABA receptor A1 in the PVN. In addition to all of the high-fat diet the animals still removed a substantial amount of normal diet. Overall, there was an increase of food removed as compared to animals that had only access to normal diet (19.7 ± 0.6 g to 23.0 ± 0.5 g, *p* < 0.001, two-tailed t-test, for the control mice, and 13.5 ± 0.6 g to 20.7 ± 0.3 g, *p* < 0.001, two-tailed t-test, for the mice without Gabra1 in the PVN, comparison of their ZT2-4 bins, Figures 4B,F). This finding was another demonstration of the power of more palatable food to override the inhibitory signals from the homeostat system, especially, when this kind of food was given during the rest phase of an animal. It has been suggested that continuous food uptake at the wrong phase, i.e., the rest phase, increased the risk of developing metabolic syndrome and obesity (Coccurello and Maccarrone 2018; Baik 2021). An interesting therapeutic approach has been developed based on these observations. Time-restricted feeding limits the uptake of food only to the activity phase (Hatori et al., 2012; Acosta-Rodriguez et al., 2017). Using these feeding conditions, animals were even protected from the unhealthy effects of high-fat diet (Chaix et al., 2019). Since the SCN are determining the time of the activity and rest phases, there appears to be a link to healthy food uptake.

Interestingly, with our simple food removal assay, we seemed to trigger another system affecting the motivation to search for food. Every time when we give the feeding cube to the animals, they started removing quite a bit of food in the time interval between ZT4 and ZT6 (Figures 1–3, 5). We suspect that this behavior was mainly curiosity driven and triggered by the recognition of the feeding cube as a source of food. Blocking the output from the SCN did not affect the appearance of this phenomenon (Figures 2B, 3B, 5B), but inhibiting the GABA receptor A1 in the PVN did (Figure 4A). The data would indicate that this recognition involves GABAergic signaling from a yet to identify brain region to the PVN, which may originate from the cerebellum (Herman et al., 2020). As anecdotal evidence, mice that received normal diet after high-fat diet considered the cube now as source of more palatable food. As consequence, on their quest for high-fat diet, they shuffled out three to four times as much food in the ZT4 to ZT6 time interval as the controls. Hence, more palatable food has a nearly addictive-like character as already described (Coccurello and Maccarrone 2018; Mendoza 2019b).

The surprising finding of this study, however, was that the SCN increased the search for food at the beginning of the activity phase. Indeed, it became apparent that by blocking the connection to the PVN, food removal was generally delayed compared to the controls (Figures 2–5). This observation fitted snuggly with one of the suspected functions of the circadian system governed by the SCN, to provide a stable phase relationship between the organs (Albrecht 2012). With functional SCN, the peripheral organs can anticipate the transition from the rest to the activity phase. In the liver, for example, the expression of certain metabolic and detoxification enzymes was slightly up-regulated at this time with the potential to super-induce these genes in the actual presence of the toxins (Gachon et al., 2006). This anticipation appeared to be so important that the circadian system of the liver had to be flexible to adapt to, for example, different light/dark conditions (Stratmann et al., 2010). Hence, food uptake at the wrong time could have unhealthy consequences for an animal.

How would the SCN motivate the search for food? It was previously demonstrated that the neurons of the SCN of mice are mainly active during the rest phase (Belle et al., 2009; Atkinson et al., 2011; Belle and Diekman 2018). However, the different subregions of the SCN could govern rhythmic output all over the circadian cycle. Detailed single-cell analysis of the SCN region revealed at least five different subregions based on their neuronal composition (Wen et al., 2020; Morris et al., 2021). Here, we focused only on two of these subregions, the Avp and the Vip subregion, which are an important output subregion and the light input subregion, respectively (Abrahamson and Moore 2001; Vosko et al., 2015; Mieda 2019; Ono et al., 2021b). The contribution of the other subregions to the motivation of food removal is currently unknown. The analysis of this contribution is interesting, because it is not yet known, how the SCN would sense the nutrition state of an animal. As speculation, the signal could come from either the Arc or intergeniculate leaflet region (Yi et al., 2006; Saderi et al., 2013), or directly from the CSF filling the third ventricle. While the SCN activates the tanycytes lining the third ventricle to facilitate glucose secretion into the CSF (Rodriguez-Cortes et al., 2022), signals could also come back from these cells to the SCN (Langlet 2014; Bolborea et al., 2020). Further analysis is clearly necessary to find the place of the SCN within the regulatory network to motivate or inhibit the search for food.

Overall, the data point to the PVN as the integrator of rhythmic food searching patterns. Many of the output fibers from the SCN, especially the Avp subregion, project to the PVN (Dai et al., 1997; Abrahamson and Moore 2001). Functional evidence for these projections exists. GABAergic signaling from the SCN to the PVN affects the glucose concentration in the blood (Kalsbeek et al., 2004). This effect is mediated by GABAergic inhibition of sympathetic pre-autonomous neurons in the PVN affecting the liver metabolism. The graft of SCN tissue into mice with lesioned SCN could restore rhythms in the PVN, indicating a humoral synchronizing signal derived from the SCN (Tousson and Meissl 2004). This signal could be related to the same signal used by the SCN to influence the Arc (Rodriguez-Cortes et al., 2022). Furthermore, light inhibition of food searching was mediated by the Avp neurons in the SCN inhibiting the oxytocin neurons in the PVN (Santoso et al., 2018), while activation of food searching was mediated by AgRP neurons in the Arc inhibiting the oxytocin neurons in the Paraventricular hypothalamus (Atasoy et al., 2012). Our data suggest that GABAergic signaling from the Avp subregion of the SCN to yet to identify targets in the PVN somehow activates the search for food (Figure 5). The importance of GABAergic signaling from the SCN to the PVN has already been described (Kalsbeek et al., 2004; Paul et al., 2020). To address the precise nature of these connections, it is probably worth to identify the cells involved by single-cell RNA sequencing.

Our data strengthen the importance of restricting food uptake to a certain time of the day, because at least two regulatory systems, the energy balancing system of the PVN and the circadian system of the SCN motivate food removal at the beginning of the activity phase. Hence, the time of food uptake appears to be important. Our findings confirm the importance of the coupling of food uptake to the activity phase, and may consequently form the base for the success of dietary approaches such as time restricted food uptake.

## Data Availability

The raw data supporting the conclusions of this article will be made available by the authors, without undue reservation.

## Supplementary material

Supplementary Figure S1 Absence of eGFP/tetanus toxin light chain activation in other Avp-expressing brain tissues. The supraoptical and paraventricular nuclei express Avp (Alexa 568, yellow) but there is no indication of eGFP (green) expression. ×40 objective (numerical aperture = 1.25, oil), a white bar measures 100 μm.

Supplementary Figure S2 Specific removal of GABA receptor A subtypes from the PVN region. **(A)** Injection of an AAV expressing Cre recombinase and eGFP into the PVN of control or *Gabra1* floxed mice. ×10 objective (numerical aperture = 0.3, dry), a white bar measures 75 μm. **(B)** Detection of Gabra1 (Cy3, magenta) in the mouse strains and colocalization with the Cre recombinase (eGFP, green). ×63 objective (numerical aperture = 1.3, glycerol), a white bar measures 15 μm. **(C)** Injection of an AAV expressing Cre recombinase and eGFP into the PVN of control or *Gabra2* floxed mice. ×10 objective (numerical aperture = 0.4, dry), a white bar measures 75 μm. **(D)** Detection of Gabra2 (Cy3, magenta) in the mouse strains and colocalization with the Cre recombinase (eGFP, green). ×63 objective (numerical aperture = 1.3, glycerol), a white bar measures 15 μm.

Supplementary Figure S3 Control for the injection of FLP recombinase into the PVN region. Detection of Cherry (red) and Avp (Alexa 568, yellow) in the PVN region of control AAV-injected mice, or mice injected with an AAV allowing retrograde transport of the FLP recombinase and Cherry. ×40 objective (numerical aperture = 1.25, oil), a white bar measures 100 μm.
