# Supplementary figures and images for: Suprachiasmatic to paraventricular nuclei interaction generates normal food searching rhythms in mice
Source: Front Physiol. 2022 Oct 6;13:909795. doi: 10.3389/fphys.2022.909795 (PMC9582613; doi:10.3389/fphys.2022.909795)

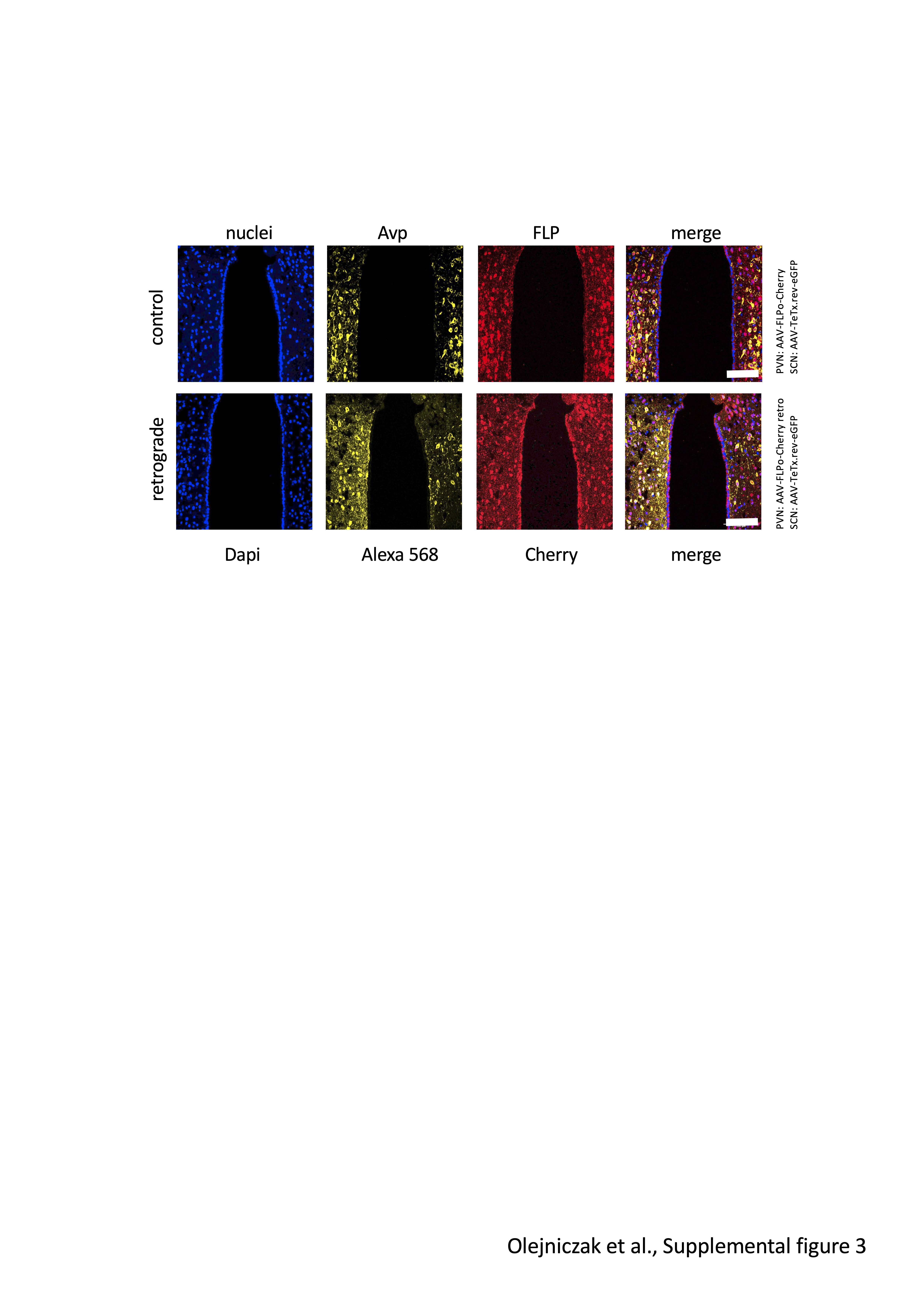

Supplement: Supplementary file 1 [file Image3.JPEG]

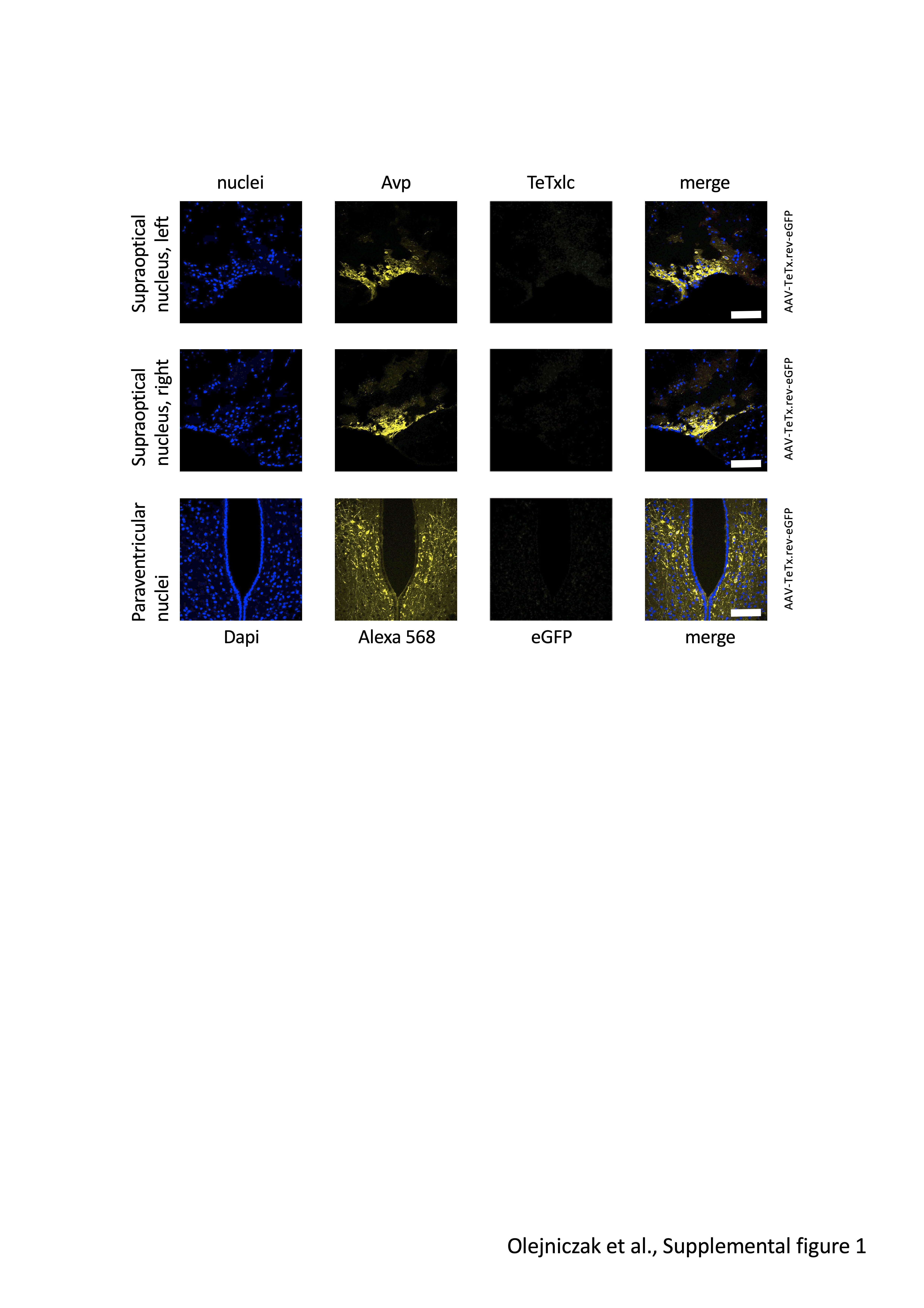

Supplement: Supplementary file 2 [file Image1.JPEG]

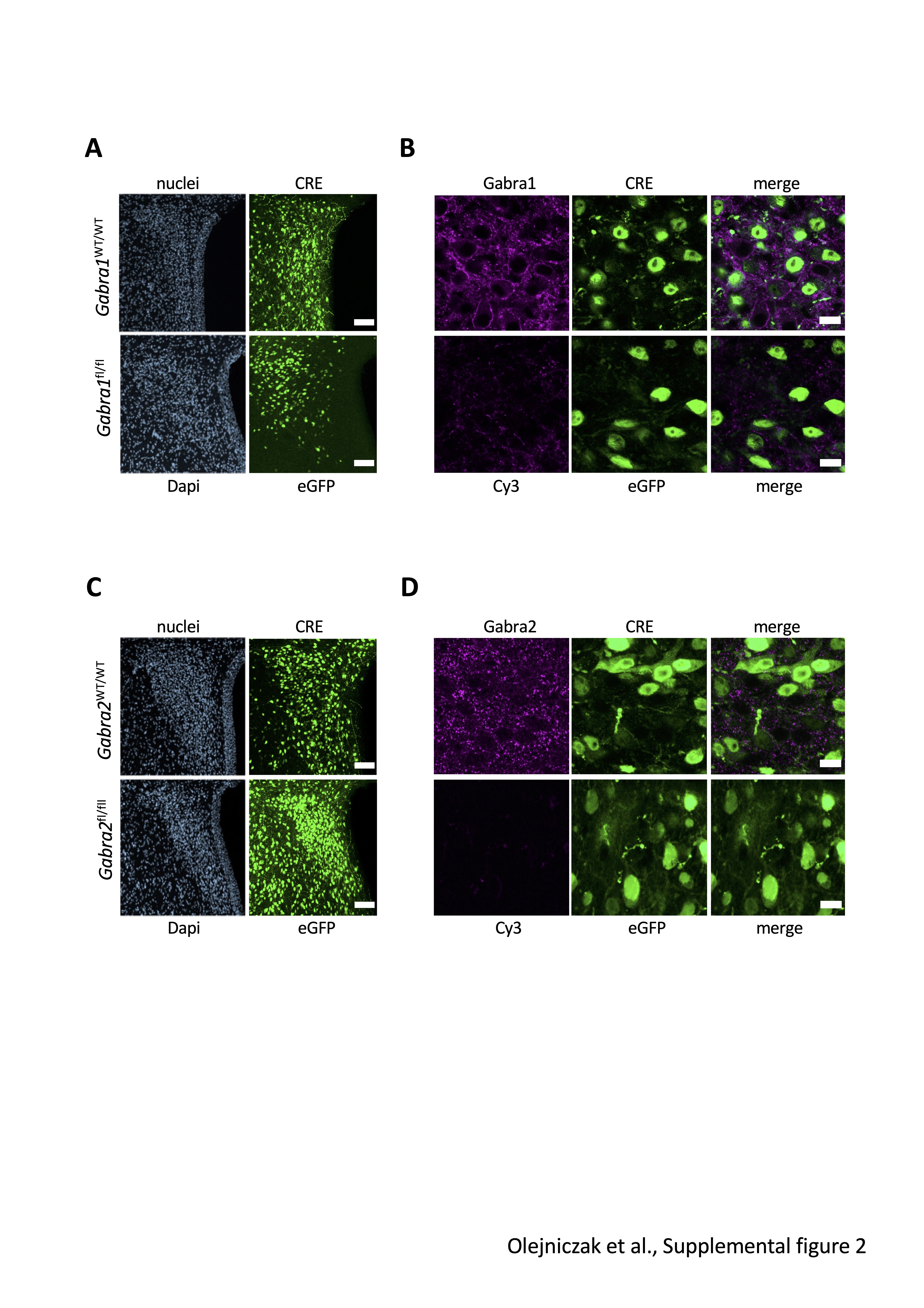

Supplement: Supplementary file 3 [file Image2.JPEG]
